# Gummy Smile: Mercado-Rosso Classification System and Dynamic Restructuring with Hyaluronic Acid

**DOI:** 10.1007/s00266-021-02169-8

**Published:** 2021-02-22

**Authors:** Jorge Mercado-García, Paula Rosso, Mar Gonzalvez-García, Jesús Colina, José Manuel Fernández

**Affiliations:** 1 Clinicas Jorge Mercado, Madrid, Spain; 2 Self Clinica, Barón de Finestrat 4, 03001 Alicante, Spain; 3 Centro médico estético Lajo-Plaza, Madrid, Spain; 4 Mar Gonzálvez-García, UCAM, Murcia, Spain; 5 Clínica Dr. Colina. Bilbao, Bilbao, Vizcaya Spain; 6 Centre Médic I D’Estética, Barcelona, Spain

**Keywords:** Gummy smile, Hyaluronic acid, Dynamic restructuring, Classification, Facial muscles

## Abstract

**Background:**

Gummy smile (GS) is a nonpathological condition causing esthetic disharmony in which an excessive amount of gingival tissue is exposed when smiling. Nowadays, there is not unanimous agreement regarding both classification and management of GS. This study aimed to present an organized and comprehensive clinical classification of the GS, as well as to discuss a therapeutic approach, with hyaluronic acid dermal fillers.

**Methods:**

This study is presenting the clinical experience of the authors regarding GS.

**Results:**

The Mercado-Rosso GS classification has into account aesthetic aspects, etiopathogenetic criteria, and functional aspects of the smile. According to Mercado-Rosso GS-classification-system, GS is divided into 3-types: Type 1, characterized by a lack of support and/or a lack of projection of the upper maxilla; Type 2, due to an imbalance between the strength (excess) and the resistance (defect) of the levator muscles; and Type 3, defined by an excessive strength of the zygomatic muscles, which causes a wide smile and an excessive visualization of the molar teeth.

**Conclusions:**

The Mercado-Rosso GS classification system is a tool that facilitates the diagnostic and therapeutic approach to the gummy smile. RD Dynamic Restructuring® constitutes a comprehensive therapeutic approach that makes reference to both the effect of the HA filler on the muscle movement and the balance between the muscle strength and the resistance of the soft tissue to be folded in different facial structures). Level of evidence: Level V.

**Supplementary information:**

The online version contains supplementary material available at (10.1007/s00266-021-02169-8)

*Level of Evidence IV* This journal requires that authors assign a level of evidence to each article. For a full description of these Evidence-Based Medicine ratings, please refer to the Table of Contents or the online Instructions to Authors www.springer.com/00266

## Introduction

The smile is a common human expression that reflects different feelings [1]. The smile is an important aesthetic component of the face and significantly impact on the perception of beauty and personality that the others have about us. Additionally, asymmetries in our face or expressions, as well as face proportions, also play an important role in the perception of beauty [2, 3].

Mimetic facial muscles (MFM) have various features that differentiate them from other skeletal muscles. The first one is the lack of any tendinous or aponeurotic intermediaries [4]. MFM are, indeed, directly attached at each end and generally originate from underlying bone surfaces and insert to the skin of the face or intermingle with other facial muscles [5].

Upper lip muscles include the zygomaticus major and minor, the levator labii superioris (LLS), the levator labii superioris alaeque nasi (LLSAN), as well as the levator anguli oris (LAO) [6, 7]. Different levator muscles pull the upper lip and the corner of the mouth upwards, while the zygomatic muscles have a diagonal action [6, 7]. The lip muscles can be divided in dilator and constrictor muscles [8]. Dilator muscles are, in turn, distributed into two layers, namely superficial and deep. The superficial layer contains seven muscles: LLSAN, LLS, zygomaticus major and minor, risorius, depressor anguli oris (DAO), and platysma [8].

**Fig. 1:**
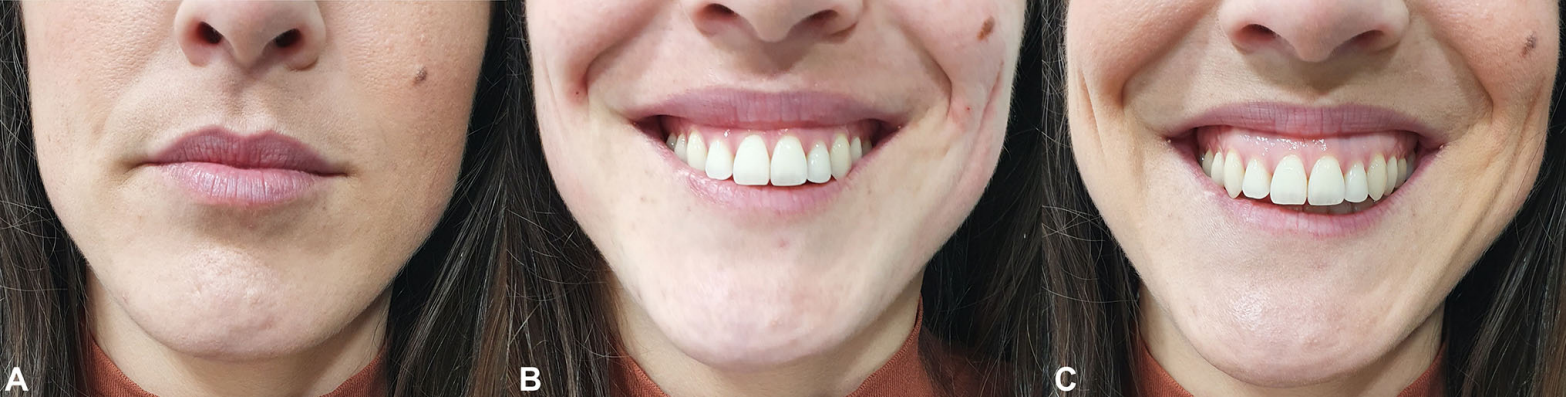
Stages in the genesis of a full smile [1]. **a** Stage 0: rest position. **b** Stage 1: upper lip elevation to the nasolabial fold. **c** Stage 2: maximum upper lip and fold elevation. Adapted from Peck et al.

**Fig. 2:**
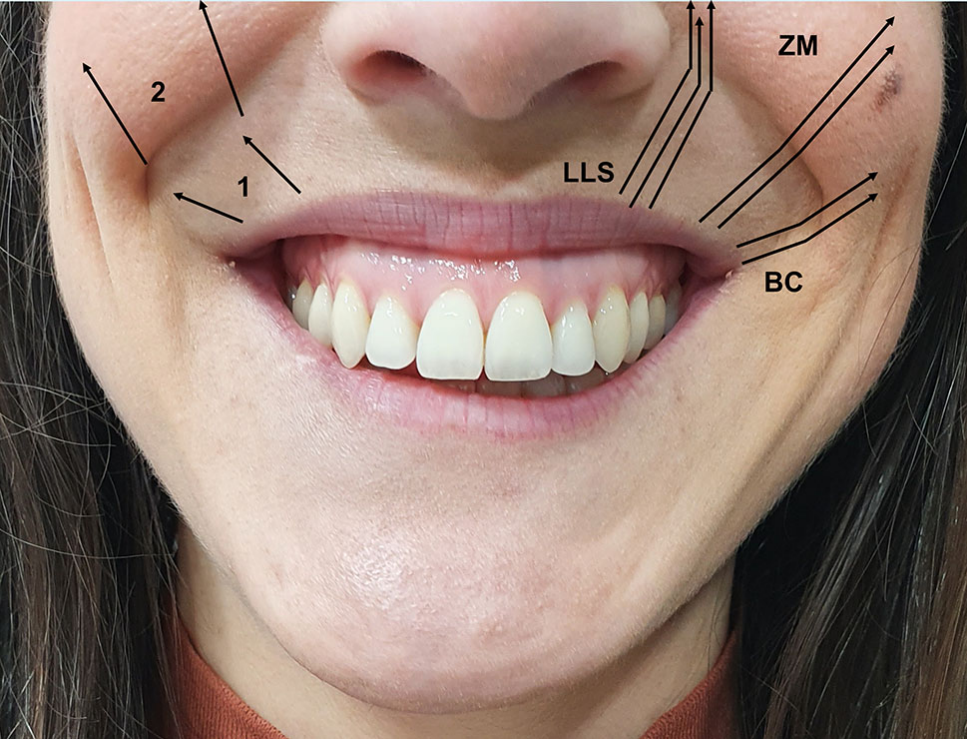
Different muscles involved during a maximum smile. The superior lip and the fold raise due to the action of three different muscle groups: The levator labii superioris (LLS), zygomaticus major (ZM), and superior fibers of the buccinator (BC)

The characteristics of the smile are determined by the interaction of the static and dynamic relationships between the dento-skeletal and soft tissue components of the face. The smile is formed in two stages (Fig. 1). During the first stage, the contraction of the levator muscles raises the upper lip to the nasolabial fold. The second stage involved further raising superiorly of the lip and the fold by three muscle groups: (1) the levator labii superior muscles of the upper lip, originating at the infraorbital region; (2) the zygomaticus major muscles; and (3) superior fibers of the buccinator (Fig. 2) [9].

Although showing a certain amount of gum (1 mm–2 mm) during a normal mile is aesthetically acceptable and in many cases imparts a youthful appearance [10–12], excessive gingival visualization during the smile has been an aesthetic problem for many patients, which can definitely affect their psychosocial behavior [13].

Perception of excessive gingival display is also subject to cultural and ethnic preferences. The quantity of gingival showed that is considered unaesthetic, or excessive, is highly subjective, and varies between males and females [14, 15], and between professionals and laypeople [16, 17]. For example, in some European countries gingival display of up to 4 mm or more is acceptable, while exposure greater than 2–3 mm is considered unsightly in the USA [18].

Gummy smile has been defined as a nonpathological condition causing esthetic disharmony in which more than 3 mm of gingival tissue is exposed when smiling [10, 19]. The GS constitutes a prevalent condition that occurs in 10.5%–29% [19, 20] of young adults, with the prevalence being higher in women [10].

The cause of the GS can be multifactorial and must be accurately diagnosed to render appropriate treatment. Factors that contribute to the GS include altered passive eruption, plaque-/drug-induced gingival enlargement, lip length, lip hypermobility, incisal wear/crown length, vertical maxillary excess, and gingival hyperplasia [21–24].

There is not a clear consensus about definition or a therapeutic approach of GS that provides predictable results, independently of its etiology.

Several classifications of GS have been previously proposed [23, 25–28], although none of them have had into consideration functional or dynamic aspects of the GS.

Regarding treatment, several treatment modalities have been used for its management.

As a general rule, GS treatment should be selected attending to its etiology. For example, orthognathic surgery may be the election technique in those cases where GS be due to vertical maxillary excess caused by excessive maxillary growth [29–31]. If GS would be due to gingival hyperplasia o altered passive eruption, orthodontic treatment using temporary anchorage devices or crown lengthening procedures should be indicated [32, 33]. Additionally, other surgical techniques have been proposed, such as muscle resection [34] and lip re-positioning [35].

The purpose of this article is twofold: (1) to present an organized and comprehensive clinical classification of the GS, which allows a therapeutic approach according to the region to treat, and not depending on its etiology, and (2) to discuss a therapeutic approach, with HA dermal fillers, that has into account not only anatomic, but also functional aspects.

## Methods

This study is presenting the clinical experience of the authors regarding GS classification and a comprehensive therapeutic approach with hyaluronic acid (HA) dermal fillers.

### Classification of gummy smile

The smile can be classified according to different parameters (Table 1). For example, depending on the lips raising direction and the muscle group involved in the smile, it is classified into three categories: The cuspid smile, the complex smile, and the commissure smile or Mona Lisa smile [9, 36, 37].

**Table 1:** Different classification system of the smile. Adapted from Rubin [36] and Londoño and Botero [37]

	Depending on the lips raising direction		
	Cuspid smile	Complex smile	Commissure smile*
Muscles involved	Participation of all the levator labii superioris	Simultaneous action of levator labii superioris and lower lip depressors	The zygomatic major muscles bring the commissures up and outwards, followed by a gradual elevation of the upper lip as in an arch shape
	According the level of consciousness		
	Voluntary smile	Static smile	Involuntary smile
Characteristics	May or may not be motivated by an emotion	Extendable and reproducible	1. Induced by gladness 2. Has a dynamic nature. 3. Expresses authentic human emotions. 4. Cannot be sustained for long periods of time
	According to gingival line localization		
	High	Medium	Low
Characteristics	The gingival line when smiling displays 100% of the anterior tooth and even a portion of the gum	The smile line exposes between 75 and 100% of the tooth	The smile line only shows 50% or less of the incisor it is considered to be a lower smile

*Also called Mona Lisa smile

### Therapeutic strategies of the gummy smile

Attending its etiology, GS correction comprises different therapeutic strategies. Sometimes, if GS is due to gingival hyperplasia o altered passive eruption, orthodontic treatment using temporary anchorage devices or crown lengthening procedures should be assessed [32, 33]. For example, orthognathic surgery is indicated in those cases of vertical maxillary excess caused by excessive maxillary growth [34, 35].

Apart from those described above, in the literature we find other surgical GS treatments such as muscle resection [28] and lip re-positioning [29].

These interventional procedures have provided good results, but they are costly, time-consuming, and their incidence of complications is high, or simply, they are not acceptable for the patients [38] as shown in Table 2.

**Table 2:** Overview of the therapeutic strategies used for treating different types of gummy smile according to the Mercado-Rosso gummy smile classification system

Type of GS	Main cause	Treated area	Main affected muscle	Type of HA	Amount of HA	Administration	Depth
Type1	Lack of structural support due to bone deficiency and/or a lack of projection of the upper maxilla	From the piriformis fossa to the midline	Orbicularis	23 mg/mL	A total of 0.6 mL of HA distributed in 12 retrograde injections (0.05 mL per application) per side	25G blunt microcannula and a fanning technique	Deep, supramuscular.
Type 2	Length–Tension relationship imbalance	Piriformis fossa (looking for the LLS)	Levator labii superioris and the levator labii superioris alaeque nasi	23 mg/mL	A total of 0.2–0.4 mL of HA per side at the piriformis fossa. A total of 0.2 mL per side at the levator labii superioris alaeque nasi. A total of 0.2 mL per side at the anterior nasal spine	25G blunt microcannula and a fanning technique	Deep, supramuscular and intramuscular
Type 3^1^	Excessive mechanical action of zygomatic muscle associated with a Type 1 or Type 2 GS	Malar area + piriformis fossa (depending on whether it is a type 1 or a type 2)	Zygomaticus major and minor	23 mg/mL 25 mg/mL	A total of 2 injections at zygomaticus major and minor (per side) (0.2 to 0.4 mL per injection additionally, the technique used for treating type 1 or type 2 GS	27G needle^2^ 25G blunt microcannula and a fanning technique	Deep, perioustium (malar area) (type 3) + Deep supramuscular, intramuscular (type2) Deep, supramuscular (type 1)

*HA* Hyaluronic acid; *LLS* Levator labii superioris

^1^In addition to treat type 3 gummy smile as a pure type 1or 2, or as a mixed type, when treating type 3 the muscular balance that it gives us The Dynamic Restructuring® on the zygomatic muscles must be sought

^2^A 27G needle is used to infiltrate malar area at periosteum level, passing through minor and major zygomatic ligaments approximately, and injecting 0.2–0.4 mL per point. Once a reduction in width smile has been observed, it is time to proceed to treat the upper lip and muscles, depending on whether gummy smile was classified as type 1 or type 2

## Results

### Mercado-Rosso classification of gummy smile

According to the Mercado-Rosso classification, gummy smile is divided into three different types: Type 1, characterized by a lack of support and/or a lack of projection of the upper maxilla. This type is defined by a thin white lip, associated with the presence of perioral wrinkles (barcode). Type 2, characterized by a deep pyriform fossa, thickness of the upper lip is slightly greater, fewer skinfolds and wrinkles, and a higher lip elevation at the areas of the 12th and 13th, as well as 22th and 23th dental pieces due to an imbalance between the strength (excess) of the levator muscles and the resistance (defect) of the soft tissue. Finally, the type 3 is defined by an excessive strength of the zygomatic muscles, which causes a wide smile and an excessive

**Fig. 3:**
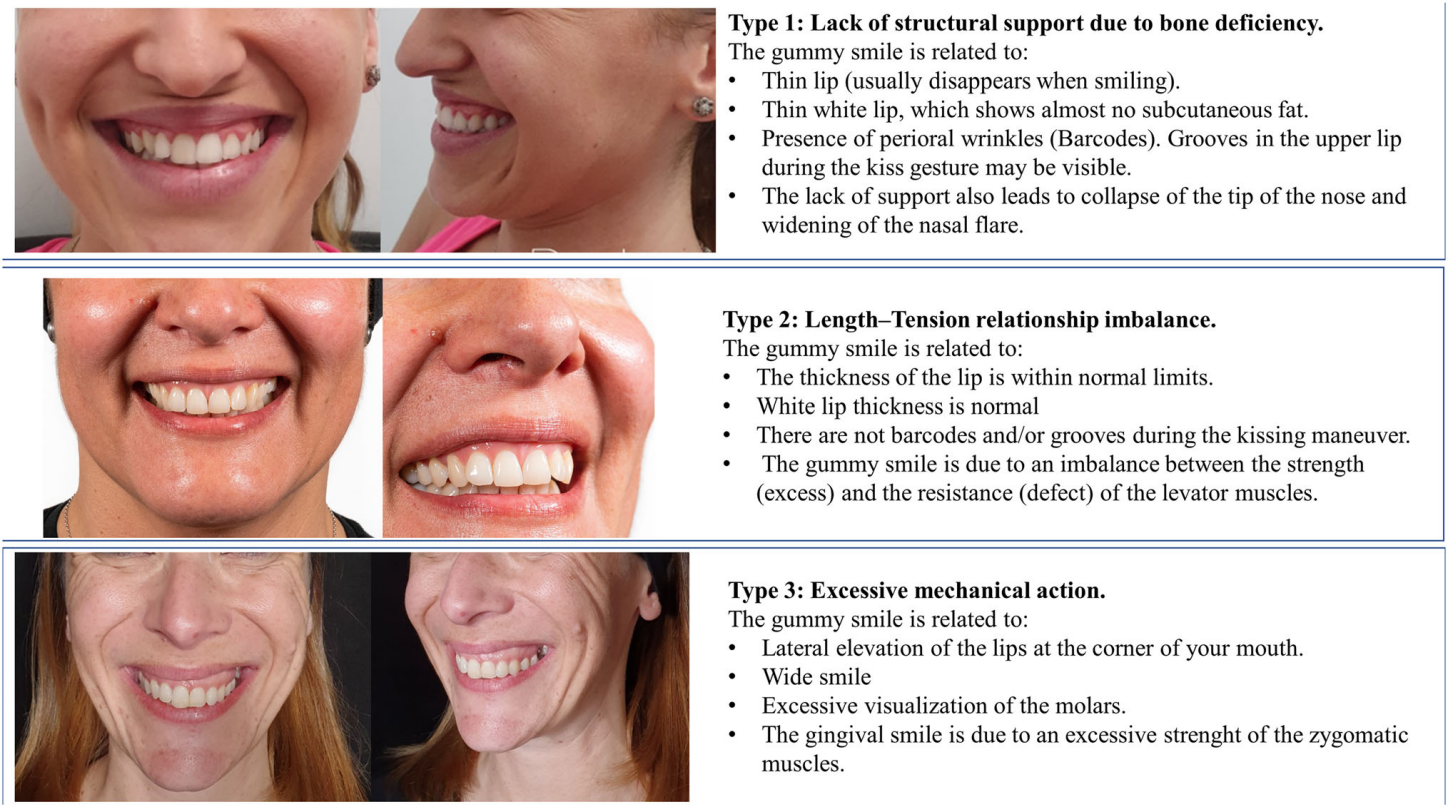
Mercado-Rosso gummy smile classification

Figure 3 shows the Mercado-Rosso gummy smile classification system.

According to the Mercado-Rosso GS classification system, there might be mixed forms (coexistence of mechanisms related to different types), which should be treated, therefore, according to a comprehensive approach.

### Treatment approach of gummy smile with hyaluronic acid fillers according to the Mercado-Rosso classification

The therapeutic approach proposed in this paper is based on the concept of RD Dynamic Restructuring®. RD Dynamic Restructuring® makes reference to the action of the HA fillers on the muscle movement, looking for balance between the muscle activity and different facial structures (bone, superficial musculoaponeurotic system, subcutaneous cellular tissue, and skin), by stretching the ligaments or increasing the resistance of the soft tissues to be folded.

#### Treatment of gummy smile type 1

As aforementioned, according to the Mercado-Rosso classification, the gummy smile Type 1 is characterized by a lack of structural support. In this type of gummy smile the treatment strategy is:

- *Administration system* Blunt microcannula (25G and 50 mm).
- *Hyaluronic acid* 23 mg/mL.
- *Depth* Deep Supramuscular and/or in a multilayer approach.
- *Total amount* 0.6 mL of HA per side, distributed in 12 retrograde injections (0.05 mL per application) per side.
- *Treated area* The whole white lip, from the entrance to piriformis fossa to the midline.

**Fig. 4:**
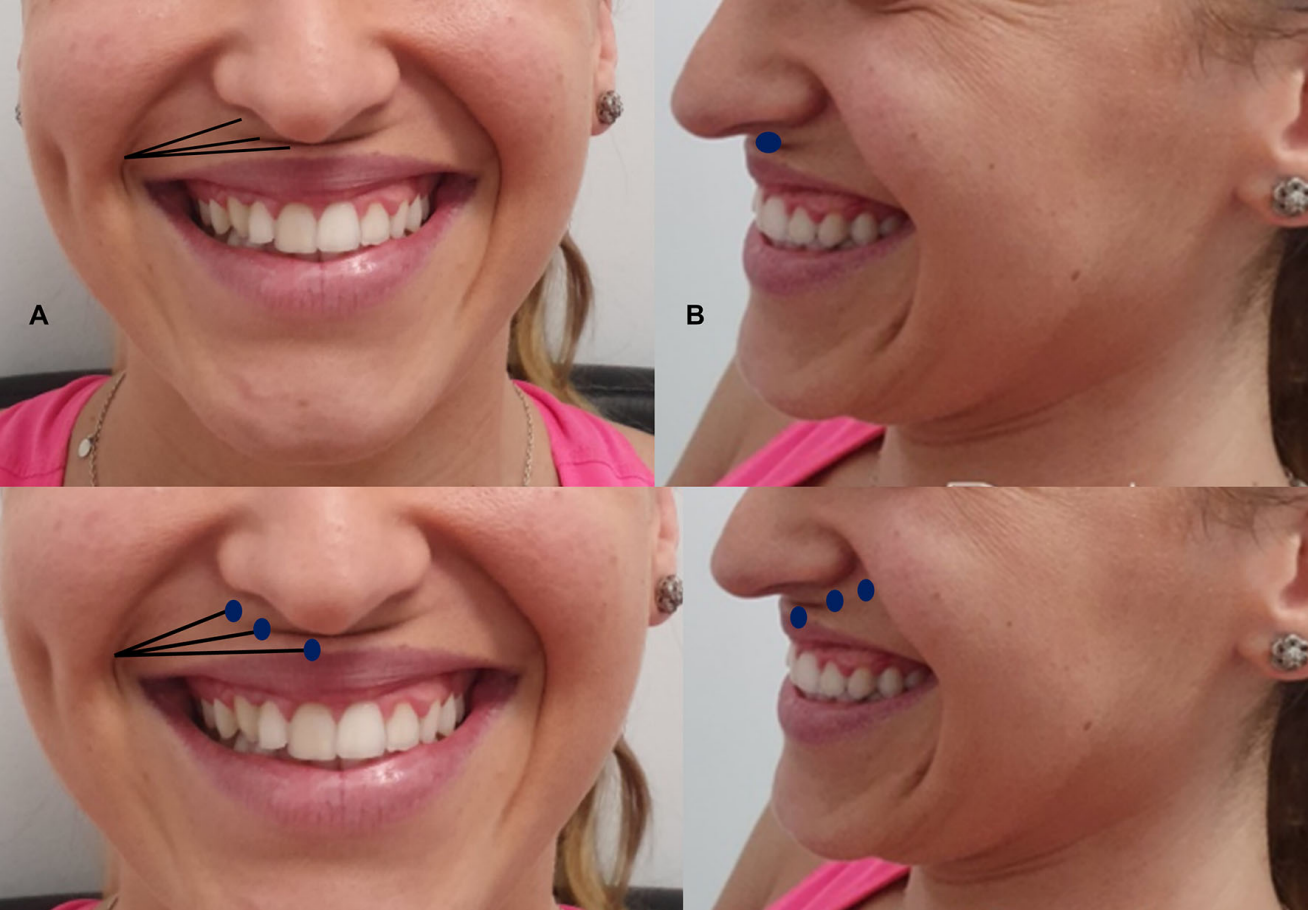
Treatment strategy of gummy smile Type 1. There is an important lack of structural support that, besides a gummy smile, causes a drop of the tip of the nose Upper image: The recommended strategy is 0.6 mL/per side of high-density hyaluronic acid (HA) filler (RHA4^®^, Teoxane, Geneve, Switzerland) administered by means fanning retrograde technique with a blunt microcannula. In this case it would be necessary to inject an additional bolus (blue ellipse) of 1 mL–2 mL of HA 23 mg/mL. Lower image: The recommended strategy is 0.6 ml/per side of high-density hyaluronic acid (HA) filler (RHA4^®^, Teoxane, Geneve, Switzerland) administered by means fanning retrograde technique with a blunt microcannula. Additionally, small boluses (blue ellipses) 0.4 mL–0.6 mL de HA 23 mg/mL at the end of each fanning retrograde administration upon reaching the central region of the white lip and circumscribed to the edges of the insertion of the nasal wings. **a** Frontal view. **b** lateral view

At approximately 5 mm of the corner of the mouth, by means a blunt microcannula (25G and 50 mm), with a retrograde fanning technique from the entry point to the piriformis fossa to the midline, 0.6 ml of HA (23 mg/mL) is injected at a supramuscular plane. The purpose is to act on the entire white lip, with the objective of providing (and/or recovering in those cases with aging changes) structural support. The objective is correcting the projection deficit and to increase the resistance of the white lip to be folded (Fig. 4).

In those cases, with a major lack of projection of the anterior nasal spine and/or premaxilla deficiency, it would be necessary to inject an additional bolus of HA (23 mg/ml) (Teosyal^®^ RHA4, Teoxane, Geneve, Switzerland) in the premaxilla area, at the projection of the anterior nasal spine at the supraperiostium level (Fig. 4). In other cases, upon reaching the central region of the white lip, it would be preferable to leave small boluses at the end of each fanning retrograde administration (circumscribed to the edges of the insertion of the nasal wings).

**Fig. 5:**
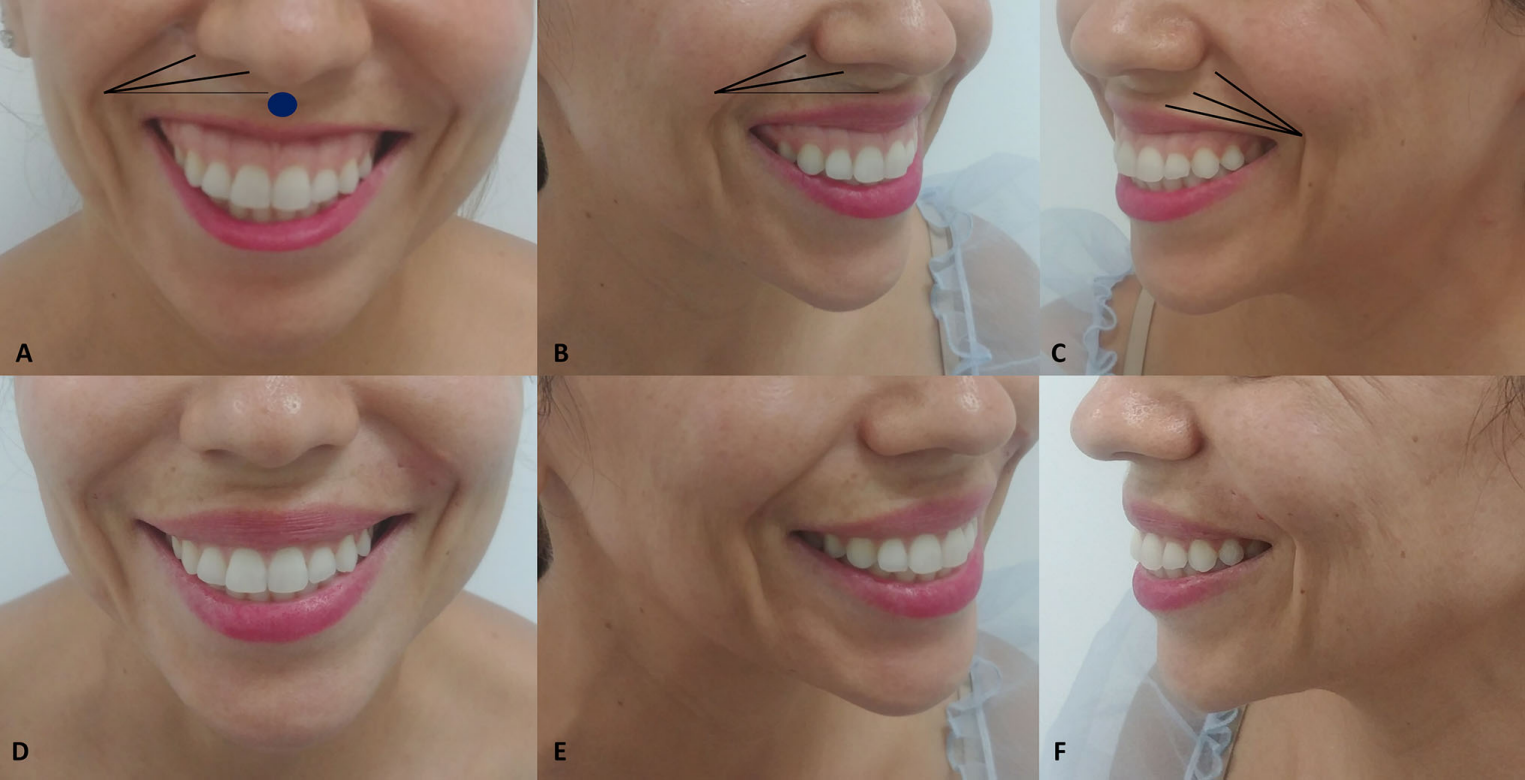
Patient with a gummy smile type 1 before (**a–c**) and after treatment (**d–f**). In this case, a retrograde fanning technique from the piriformis fossa to the midline, by means a blunt microcannula (25G and 50 mm), was used to inject 0.6 ml/side of HA (23 mg/ml) (RHA4®, Teoxane, Geneve, Switzerland) in a supramuscular plane. After treatment (D, E, and F images), it is possible to see how the RD Dynamic Restructuring^®^ technique has created a structural support and the white lip was enhanced

Figure 5 shows a patient with a gummy smile type 1 before and after RD Dynamic Restructuring^®^ with a HA filler (23 mg/ml). After treatment (Fig. 5 D, E, and F images), it is possible to see how the RD Dynamic Restructuring^®^ technique has created a structural support and the white lip was enhanced (Fig. 5).

#### Treatment of gummy smile type 2

The type 2 gummy smile is mainly defined by an unbalanced activity of the levator muscles. The recommended treatment strategy is:

- Administration system: Blunt microcannula (25G and 50 mm).
- Hyaluronic acid: 23 mg/mL.
- Depth: Supramuscular /intramuscular
- Total amount:
  - A total of 0.2–0.4 mL of HA per side at the piriformis fossa.
  - A total of 0.2 mL per side at the levator labii superioris alaeque nasi.
  - A total of 0.1 mL per side at the anterior nasal spine.

- Patient, can also have type 1 in these cases:

- Treated area Piriformis fossa (looking for the Levator labii superioris muscle).

The injection is administered at approximately 5 mm of the corner of the mouth, by means a blunt microcannula (25G and 50 mm), with a fanning technique and looking, at the piriformis fossa, for a deep plane superficial to levator labii superioris. It is recommended, before to start the HA administration, that the patient gesticulates for determining the blunt shifting.

Once the levator labii superioris muscle has been located, we proceed to inject a bolus of 0.2 to 0.4 mL of a crosslinked HA filler (23 mg/mL), with the goal of modulate the muscle activity. As a second step, the canula should be medially slide, looking for a parallel point, almost under the nasal wing insertion, which allows to limit the strength of contraction of the levator labii superioris alaeque nasi.

**Fig. 6:**
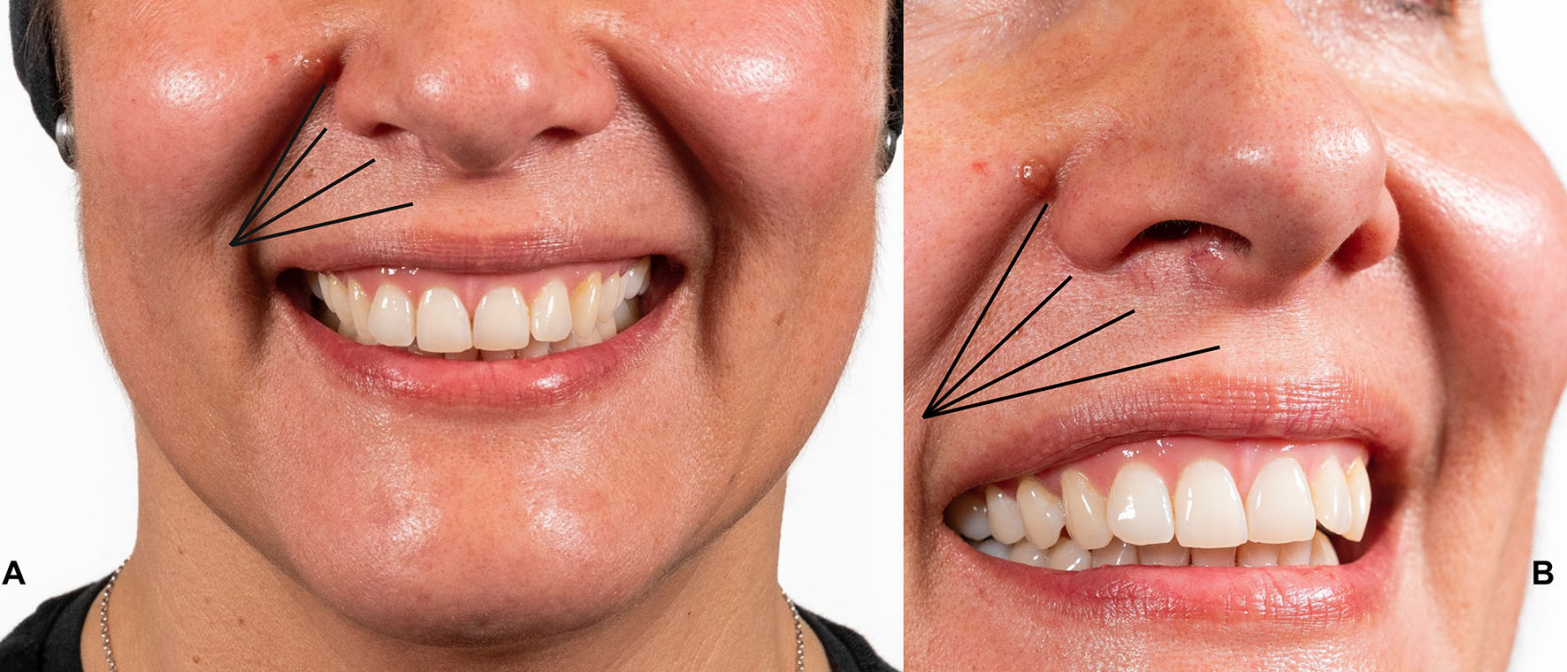
Treatment strategy of gummy smile Type 2. The recommended strategy is a total of 0.6 mL–0.8 mL/per side of a high-density hyaluronic acid (HA) filler (RHA4®, Teoxane, Geneve, Switzerland) administered at the piriformis fossa (0.2 mL–0.4 mL/side); at the levator labii superioris alaeque nasi (0.2 mL/side); and at the anterior nasal spine (0.2 mL/side). **a** Frontal view. **b** Lateral view

At this point, approximately 0.1 mL of 23 mg/ml HA filler should be injected. Finally, sliding the blunt microcannula to the nasal spine, but without touching it, 0.2 mL of 23 mg/ml HA filler needs to be placed on the depressor septi nasi muscle (Fig. 6).

Once the effect of RD Dynamic Restructuring® on the gingival smile has been observed, it is time to proceed to treat the underlying Type 1 gummy smile, as appropriate.

#### Treatment of gummy smile type 3

There is an overactivity of the zygomatic muscles.

The recommended treatment strategy is:

- Administration system: Needle (27G and 30 mm).
- Hyaluronic acid: 25 mg/mL.
- Depth: Periosteum.
- Total amount: 0.4 mL–0.8 mL of HA per side, distributed in 2 boluses (0.2 mL–0.4 mL/per bolus/per side).
- Treated area: Malar region.
- Patient, can also have type 1 o 2 characteristics, in these cases:
  - Type 1 or Type 2 therapeutic strategy (depending on the diagnosis).

**Fig. 7:**
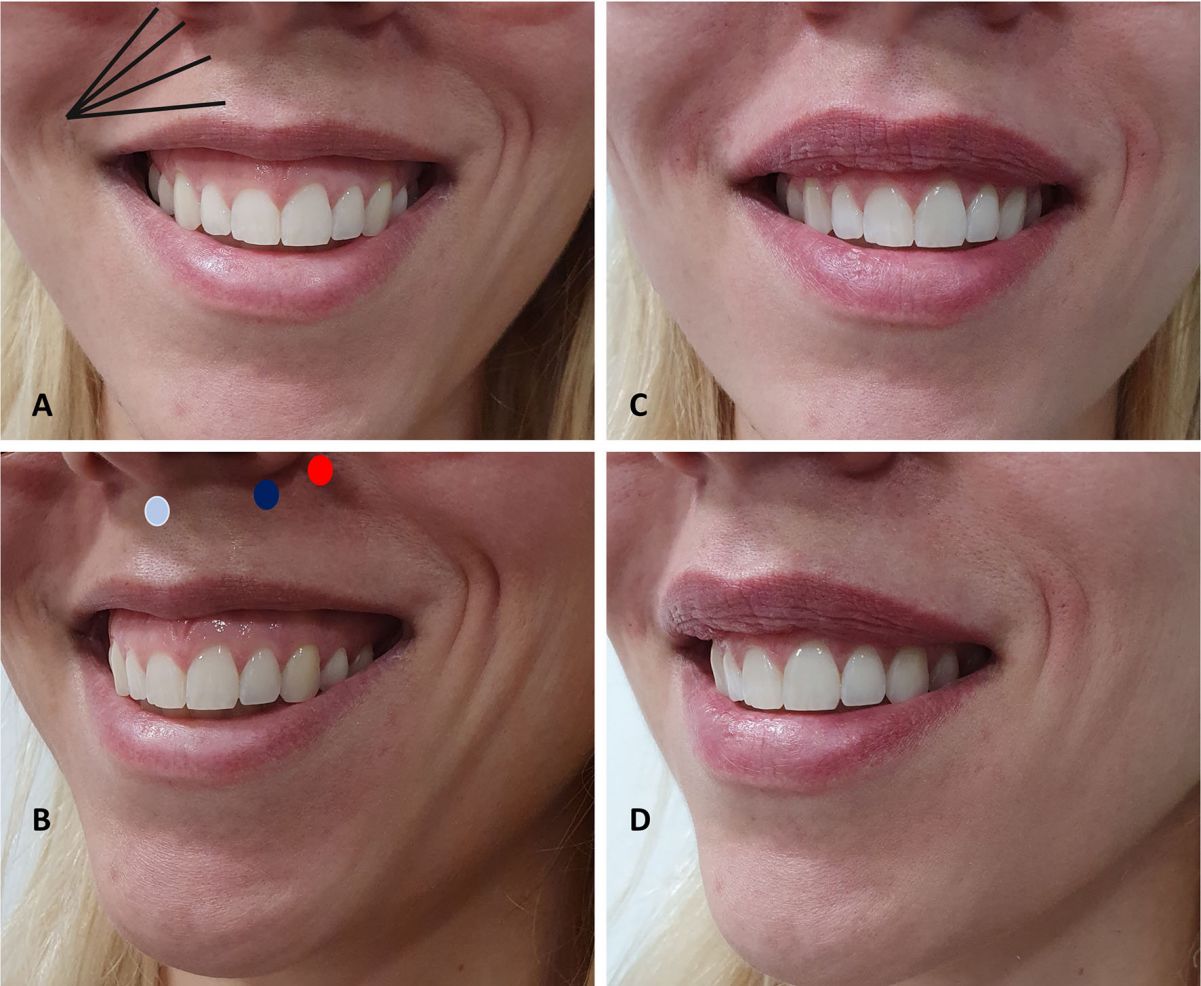
Patient with a gummy smile Type 2. The patient was treated with a bolus of 0.6 ml (per side) of a 23 mg/mL hyaluronic acid filler (RHA4®, Teoxane, Geneve, Switzerland) administered by using a fanning retrograde technique with a 25G blunt microcannula in the white lip (black lines); a bolus of 0.3 mL/per side of HA 23 mg/ml administered at the piriformis fossa (red ellipse) with a 25G blunt microcannula; 0.1 ml/per side of HA 23 mg/mL at the levator labii superioris alaeque nasi (dark blue ellipse); and 0.1mL/per side of HA 23 mg/mL at the anterior nasal spine (between nasal spine and orbicular) (light blue ellipse). Vermillion was not treated. **a** Frontal view before treatment; **b** Lateral view before treatment; **c** Frontal view after treatment; **d** Lateral view after treatment

RD Dynamic Restructuring® of zygomatic muscles is done by means a 27G and 30 mm needle, which is injected in the malar region, at the periosteum level, passing through the zygomaticus ligaments. Two injection points with 0.2 to 0.4 mL per injection point of a 25 mg/mL HA filler (Ultradeep^®^, Teoxane, Geneve, Switzerland) should be administered at the malar region (a total of 0.4–0.8 mL per side). Once the effect of RD Dynamic Restructuring® on the gingival smile has been observed, we will proceed to treat the underlying Type 1 or Type 2 gummy smile, as corresponding (Fig. 7).

## Discussion

**Fig. 8:**
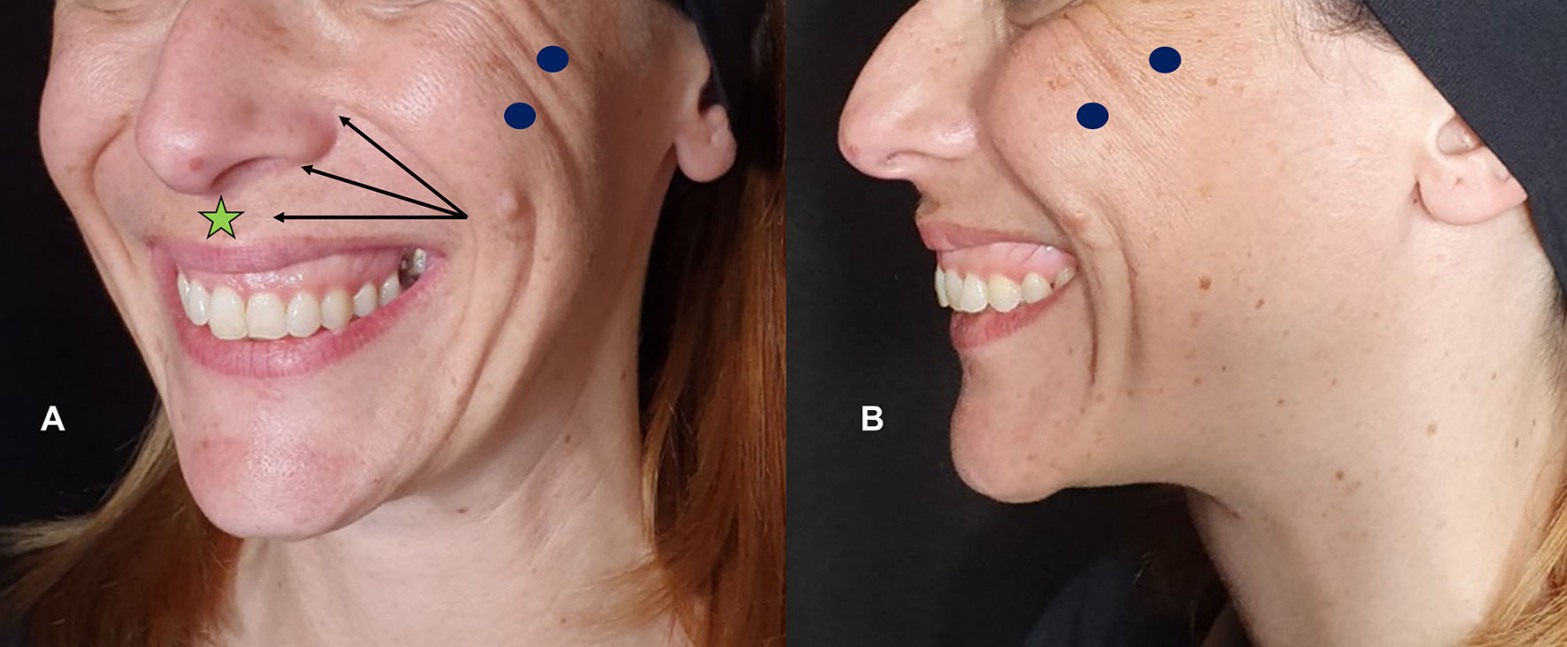
Treatment strategy of gummy smile Type 3. This patient combines a Type 1 gummy smile with an overactivity of the zygomaticus muscles. Besides the treatment of the Type 1 gummy smile (see Fig. 4), this patient needed a total amount of 0.4–0.8 mL of 25 mg/mL HA per side (Ultradeep®, Teoxane, Geneve, Switzerland) distributed in 2 boluses (0.2 mL–0.4 mL/per bolus/per side) (blue ellipse). This patient required an additional bolus of (blue ellipse) of 1 mL–2 mL of HA 23 mg/mL (RHA4®, Teoxane, Geneve, Switzerland) (green star)

**Fig. 9:**
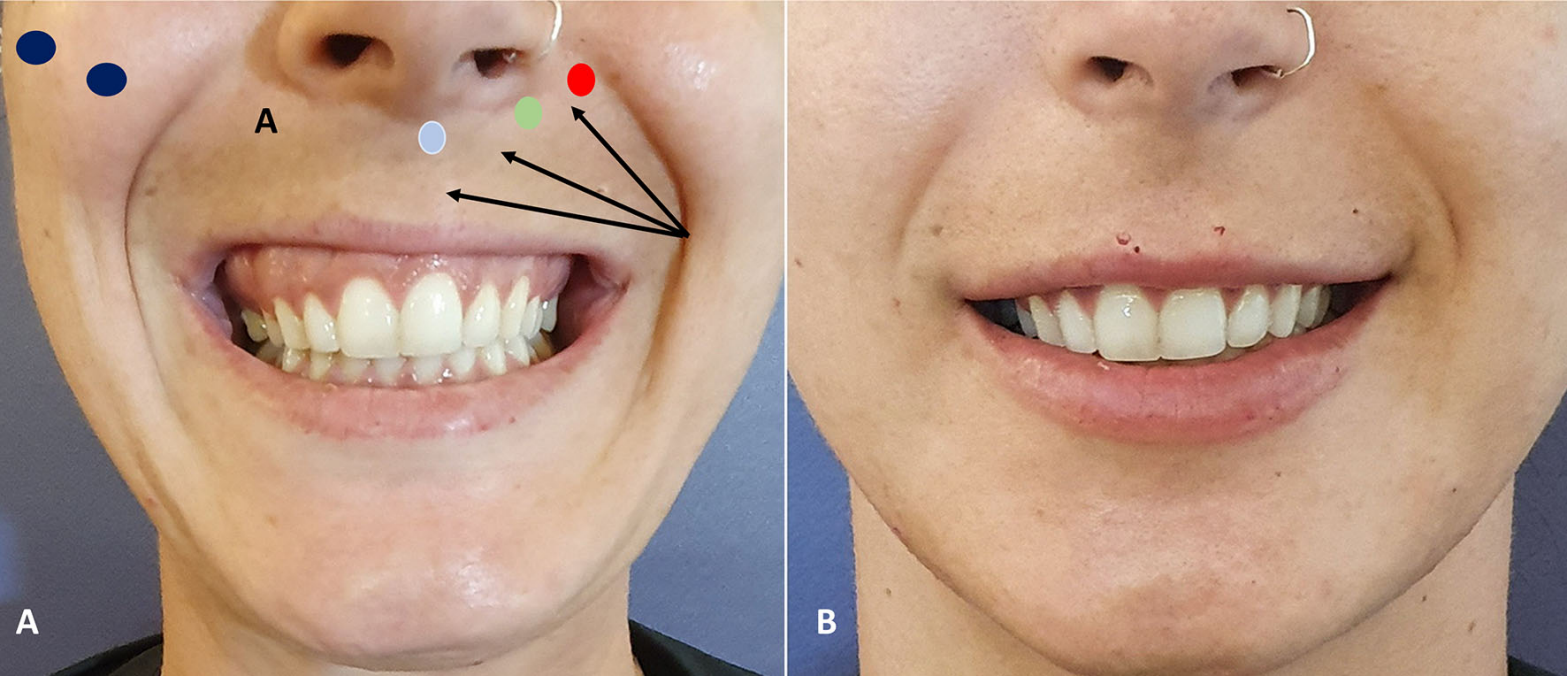
Patient with gummy smile Type 3. The patient was treated with two parallel bolus of 0.3mL (per side) of a 25 mg/mL hyaluronic acid (HA) filler (Ultradeep®, Teoxane, Geneve, Switzerland) with needle in the zygomatic ligament at the malar region (blue ellipses), bolus of 0.3 mL of HA 23 mg/mL (RHA4®, Teoxane, Geneve, Switzerland) at the piriformis fossa (red ellipse) with a 25G blunt microcannula; 0.1 mL/per side of HA 23 mg/mL at the levator labii superioris alaeque nasi (green ellipse); and 0.1mL/per side of HA 23 mg/mL at the anterior nasal spine (between nasal spine and orbicular) (light blue ellipse); and 0.4 mL of HA 23 mg/mL by using a fanning retrograde technique with a 25G blunt microcannula in the white lip (black arrows). Vermillion was not treated. **a** Frontal view before treatment. **b** Frontal view after treatment

Beauty is seen as a highly subjective feeling that results from individual factors such as sex, race, education and personal experiences, as well as social factors such as the environment and the media, which has been increasingly responsible for globalizing the concept of beauty [39] as shown in Figs. 8 and 9.

Having into consideration the differences in aesthetic perceptions and the fact that treatment goals of aesthetic specialists may not coincide with the patients’ expectations, it is crucial that the aesthetic specialist not only understand the patients concerns, but also provide objective and achievable expectations.

Moreover, when speaking about facial mimetic muscles it is important to focus not only on treatment results at rest, but also in movement.

Different classifications of GS have been proposed, although none of them have been focus in provide a comprehensive approach to the problem [23, 25–28]. Two of these classifications deal with younger patients [25, 26], while the other one had into account the area of gingival exposure and the respective muscles involved, but focusing on botulinum toxin injection [27]. Additionally, Pavone et al. [28] adapted the classification proposed by Monaco et al. [25] to adults, but their classification was based upon etiopathogenetic criteria. Finally, a simple classification system was proposed by Chu et al., who established several degrees of severity depending on the amount of gingival display at smile: level I if gingival display is between 2 and 4 mm; level II if 4–8 mm of gingiva are displayed; and level III if showing more than 8 mm of gingiva [23].

The Mercado-Rosso gummy smile classification has into account not only aesthetic aspects of the smile (quantity of gingival display at smile) or etiopathogenetic criteria, but also functional aspects of the smile, such as different group of muscles involved or the presence of potential imbalance in the relationship between the length, tension, and strength of those muscles.

Additionally, the Mercado-Rosso GS classification system allows professionals to identify not only the main functional and/or anatomic cause of the GS, but also looks for different subjacent causes that may vary the therapeutic approach.

Several therapeutic modalities have been proposed for the correction of gummy smile, both invasive [29–35] and non- or minimally invasive [40–45]. However, invasive procedures have been associated with high morbidity [38]. Therefore, options that reduce invasiveness, risks, and recovery time while still being effective are an appealing alternative.

Over the past several years, the demand for minimally invasive aesthetic procedures has grown exponentially [46]. Additionally, fillers increased by 11.6% in 2018, with a total of 3,729,833 hyaluronic acid (HA) procedures performed worldwide.

It has been described GS management with toxin in order to relax muscles hyperactivity. Botulinumtoxin A injections represent a minimally invasive option for treating GS [38, 42–45]. However, the injection of botulinumtoxin, despite being a simple and safe procedure, has a short-time limited effect and, in some cases, may be associated with ptosis or lengthening of the upper lip and asymmetry of the smile, with the subsequent unaesthetic effects [43]; moreover, it can't be removed in case of unpleasant results. Although it needs to be assessed, it might be considered the possibility to combine botulinumtoxin with HA fillers, particularly in some selected cases with a GS type 2 or 3 of the Mercado-Rosso classification.

HA fillers has been proposed as a minimally invasive therapeutic approach for treating GS [40, 41]. The effectiveness of the HA fillers on modulating the activity of the muscles has been previously described by de Maio [41]. He proposed the possibility that the HA fillers can mechanically alter muscle contraction, by either facilitating or blocking their action [41].

Moreover, there is increasing evidence suggesting that HA dermal fillers can be injected into the muscle to create a mechanical obstacle to muscle action, which may be a viable alternative for treating GS [40, 41, 47].

HA is a natural high molecular weight, belonging to the glycosaminoglycan family, which due to its physicochemical properties is capable to contain up to 1000-fold more water than its own weight [48, 49]. HA fillers have been widely used in many aesthetic procedures with good results [46, 49, 50].

Different manufacturing related factors, such as HA concentration, polymer chain length, crosslinking degree, or cross-linking technology, impact significantly on different filler properties, such as requisite needle size; particle size; duration; extrusion force; and elastic Modulus (G'), which will critically influence product selection and indication [49–54].

Among the different factor aforementioned, crosslinking is essential to slow down the enzymatic degradation rate of the HA by endogenous hyaluronidase and therefore to prolong the product’s half-life [55]. During a classical crosslinking reaction, the HA chains are partially degraded, which makes them lose part of their rheological properties [54, 55]. In order to counterbalance this degradation, a higher crosslinking rate (5‐10 %) is required, which supposes a greater rigidity of the HA filler [56].

Numerous HA fillers are available, with different characteristics [49–54]. One of the latest generation of fillers was created with a patented “preserved network” technology (Teosyal^®^ RHA, Teoxane, Geneve, Switzerland), which utilizes a proprietary to better protect the length of HA chains from degradation and optimize the degree of crosslinking. The "Preserved network"^®^ technology maintains the natural mobile interactions within the HA chains, which contributes to create a 3D network that is reinforced with anchor points with only limited amounts of 1,4-butanediol diglycidyl ether (BDDE) (1.9‐4%) [57]. As a consequence, the HA fillers produced with this technology are resilient, instead of being quite static [57], which is an essential characteristic during movement. The Teosyal® range due to its great variety and versatility, meets the requirements for treating GS, while maintaining the naturalness of facial expressions in motion.

For performing RD Dynamic Restructuring^®^ of GS, we recommended two different HA fillers of the Teosyal^®^ range: RHA4^®^ and Ultradeep^®^. Teosyal RHA4^®^ is a crosslinked HA (23 mg/mL) filler with a BDDE crosslinking of the 4.0%, which lends it a good resistance/elasticity relationship [57]. It is especially indicated in the GS type 1 and 2. Additionally, Teosyal Ultra Deep^®^ combines a high amount of HA (25 mg/mL) with a high elastic modulus (G') and high cohesivity [57]. Their characteristics make it the ideal product for RD Dynamic Restructuring^®^ of the overactivity of the zygomaticus muscles of the GS Type 3.

As a limitation, we should mention that our treatment strategy only addressed information about a specific family of HA fillers. RD Dynamic Restructuring® refers to the effect of HA fillers on muscle activity and on the resistance of the tissues to be folded. To look for balance between muscle activity, different facial structures, and filler rheological characteristics is, therefore, crucial for achieving the desired aesthetic results. Although there is no reason to suppose that other types of HA fillers cannot be used, they need to have specific physical and rheological properties for obtaining optimal aesthetic results.

The Teosyal range, due to their physical and rheological properties, is able to withstand stress forces and to adapt to the dynamic requirements of the treated zone [58].

## Conclusions

The Mercado-Rosso GS classification system is a tool that facilitates the diagnostic and therapeutic approach to the gummy smile. In most cases, the treatment maintains 70-80% of its effectiveness after 10 months of its administration. We recommend a first retreatment session after 10 months of the first treatment and a second one 18 months after the first retreatment session. Beyond second retreatment, it will be on-demand treatment.

We are aware that this paper represents a first step. The Mercado-Rosso GS classification system should be validated and different treatment approaches need to be evaluated in a cohort of patients, if possible, in multicenter studies and by different groups.

Despite these issues, the current paper provides a valuable information to those specialists who want to start with the treatment of the gummy smile.

## Supplementary information

Supplementary file 1

## References

[1] Passia N, Blatz M, Strub JR (2011). Is the smile line a valid parameter for esthetic evaluation? a systematic literature review. Eur J Esthet Dent.

[2] Beall AE (2007). Can a new smile make you look more intelligent and successful?. Dent Clin North Am.

[3] Ker AJ, Chan R, Fields HW, Beck M, Rosenstiel S (2008). Esthetics and smile characteristics from the layperson's perspective: a computer-based survey study. J Am Dent Assoc.

[4] Micheli-Pellegrini V (2011). About muscle insertions in man (Proposal for a new nomenclature of striated muscle). Acta Otorhinolaryngol Ital.

[5] von Arx T, Nakashima MJ, Lozanoff S (2018). The face – a musculoskeletal perspective. A Lit Rev Swiss Dent J.

[6] Hur MS, Hu KS, Park JT, Youn KH, Kim HJ (2010). New anatomical insight of the levator labii superioris alaeque nasi and the transverse part of the nasalis. Surg Radiol Anat.

[7] Hur MS, Youn KH, Hu KS, Song WC, Koh KS, Fontaine C, Kim HJ (2010). New anatomic considerations on the levator labii superioris related with the nasal ala. J Craniofac Surg.

[8] Olszewski R, Liu Y, Duprez T, Xu TM, Reychler H (2009). Three-dimensional appearance of the lips muscles with three-dimensional isotropic MRI: in vivo study. Int J Comput Assist Radiol Surg.

[9] Ackerman MB, Ackerman JL (2002). Smile analysis and design in the digital era. J Clin Orthod.

[10] Peck S, Peck L, Kataja M (1992). The gingival smile line. Angle Orthod.

[11] Peck S, Peck L (1995). Selected aspects of the art and science of facial esthetics. Semin Orthod.

[12] Arnett GW, Bergman RT (1993). Facial keys to orthodontic diagnosis and treatment planning. Part I Am J Orthod Dentofacial Orthop.

[13] Jacobs PJ, Jacobs BP (2013). Lip repositioning with reversible trial for the management of excessive gingival display: a case series. Int J Periodontics Restorative Dent.

[14] Geron S, Atalia W (2005). Influence of sex on the perception of oral and smile esthetics with different gingival display and incisal plane inclination. Angle Orthod.

[15] Flores-Mir C, Silva E, Barriga MI, Lagravere MO, Major PW (2004). Lay person's perception of smile aesthetics in dental and facial views. J Orthod.

[16] Kokich VO, Kiyak HA, Shapiro PA (1999). Comparing the perception of dentists and lay people to altered dental esthetics. J Esthet Dent.

[17] Ioi H, Nakata S, Counts AL (2010). Influence of gingival display on smile aesthetics in Japanese. Eur J Orthod.

[18] Armitage GC (1999). Development of a classification system for periodontal diseases and conditions. Ann Periodontol.

[19] Dong JK, Jin TH, Cho HW, Oh SC (1999). The esthetics of the smile: a review of some recent studies. Int J Prosthodont.

[20] Tjan AH, Miller GD, The JG (1984). Some esthetic factors in a smile. J Prosthet Dent.

[21] Garber DA, Salama MA (2000). The aesthetic smile: diagnosis and treatment. Periodontol.

[22] Robbins JW (1999). Differential diagnosis and treatment of excess gingival display. Pract Periodontics Aesthet Dent.

[23] Chu SJ, Karabin S, Mistry S (2004). Short tooth syndrome: diagnosis, etiology, and treatment management. J Calif Dent Assoc.

[24] Silberberg N, Goldstein M, Smidt A (2009). Excessive gingival display etiology, diagnosis, and treatment modalities. Quintessence Int.

[25] Monaco A, Streni O, Marci MC, Marzo G, Gatto R, Giannoni M (2004). Gummy smile: clinical parameters useful for diagnosis and therapeutical approach. J Clin Pediatr Dent.

[26] Wu H, Lin J, Zhou L, Bai D (2010). Classification and craniofacial features of gummy smile in adolescents. J Craniofac Surg.

[27] Mazzuco R, Hexsel D (2010). Gummy smile and botulinum toxin: a new approach based on the gingival exposure area. J Am Acad Dermatol.

[28] Pavone AF, Ghassemian M, Verardi S (2016). Gummy smile and short tooth syndrome—part 1: etiopathogenesis, classification, and diagnostic guidelines. Compend Contin Educ Dent.

[29] Fowler P (1999). Orthodontics and orthognathic surgery in the combined treatment of an excessively “gummy smile”. New Zealand Dent J.

[30] Shimo T, Nishiyama A, Jinno T, Sasaki A (2013). A case of maxillary protrusion and gummy smile treated by multi-segmental horseshoe le fort I osteotomy. Acta Med Okayama.

[31] Nishiyama A, Ibaragi S, Yoshioko N, Shimo T, Sasaki A (2017). Case of maxillary protrusion and gummy smile treated by multi-segmental horseshoe le fort I osteotomy. Int J Oral Maxillofac Surg.

[32] Kim TW, Kim H, Lee SJ (2006). Correction of deep overbite and gummy smile by using a mini-implant with a segmented wire in a growing class II division 2 patient. Am J Orthod Dentofac Orthop.

[33] Roshna T, Nandakumar K (2005). Anterior esthetic gingival depigmentation and crown lengthening: report of a case. J Contemp Dent Pract.

[34] Ishida L, Ishida LC, Ishida J, Grynglas J, Alonso N, Ferreira MC (2010). Myotomy of the levator labii superioris muscle and lip repositioning: a combined approach for the correction of gummy smile. Plast Reconstr Surg.

[35] Gaddale R, Desai SR, Mudda JA, Karthikeyan I (2014). Lip repositioning. J Indian Soc Periodontol.

[36] Rubin LR (1974). The anatomy of a smile: its importance in the treatment of facial paralysis. Plast Reconstr Surg.

[37] Londoño MA, Botero P (2012). The smile and its dimensions. Rev Fac Odontol Univ Antioq.

[38] Mangano A, Mangano A (2012). Current strategies in the treatment of gummy smile using botulinum toxin type A. Plast Reconstr Surg.

[39] Bolas-Colvee B, Tarazona B, Paredes-Gallardo V, Arias-De Luxan S (2018). Relationship between perception of smile esthetics and orthodontic treatment in Spanish patients. PLoS ONE.

[40] Hsien-Li Peng P, Peng JH (2019). Treating the gummy smile with hyaluronic acid filler injection. Dermatol Surg.

[41] de Maio M (2018). Myomodulation with Injectable Fillers: an innovative approach to addressing facial muscle movement. Aesthetic Plast Surg.

[42] Polo M (2005). Botulinum toxin type A in the treatment of excessive gingival display. Am J Orthod Dentofacial Orthop.

[43] Pedron IG, Mangano A (2018). Gummy smile correction using botulinum toxin with respective gingival surgery. J Dent (Shiraz).

[44] Dym H, Pierre R (2020). Diagnosis and treatment approaches to a "gummy smile". Dent Clin North Am.

[45] Carruthers J, Carruthers A (2000). Botox treatment for expressive facial lines and wrinkles. Curr Opin Otolaryngol Head Neck Surg.

[46] Authors no listed. The international study on aesthetic/cosmetic procedures performed in 2018. Available in: https://www.isaps.org/medical-professionals/isaps-global-statistics/ Last accessed September 21, 2020.

[47] Diaspro A, Cavallini M, Piersini P, Sito G (2018). Gummy smile treatment: proposal for a novel corrective technique and a review of the literature. Aesthet Surg J.

[48] Laurent TC, Fraser JRE (1992). Hyaluronan. FASEB J.

[49] Gutowski KA (2016). Hyaluronic acid fillers: science and clinical uses. Clin Plast Surg.

[50] Mansouri Y, Goldenberg G (2015). Update on hyaluronic acid fillers for facial rejuvenation. Cutis.

[51] Stocks D, Sundaram H, Michaels J, Durrani MJ, Wortzman MS, Nelson DB (2011). Rheological evaluation of the physical properties of hyaluronic acid dermal fillers. J Drugs Dermatol.

[52] Segura S, Anthonioz L, Fuchez F, Herbage B (2012). A complete range of hyaluronic acid filler with distinctive physical properties specifically designed for optimal tissue adaptations. J Drugs Dermatol.

[53] Greene JJ, Sidle DM (2015). The hyaluronic acid fillers: current understanding of thetissue device interface. Facial Plast Surg Clin North Am.

[54] Micheels P, Sarazin D, Tran C, Salomon D (2016). Effect of different crosslinking technologies on hyaluronic acid behavior: a visual and microscopic study of seven hyaluronic acid gels. J Drugs Dermatol.

[55] Kablik J, Monheit GD, Yu L, Chang G, Gershkovich J (2009). Comparative physical properties of hyaluronic acid dermal fillers. Dermatol Surg.

[56] Pomarède N (2009). Injection d'acide hyaluronique [Hyaluronic acid injection]. Ann Dermatol Venereol.

[57] Teoxane data on file. Available in: https://www.teoxane.com/b2b/products/teosyalr-products/dermal-fillers/preserved-network-technology Last accessed September 21, 2020.

[58] Santoro S, Russo L, Argenzio V, Borzacchiello A (2011). Rheological properties of cross-linked hyaluronic acid dermal fillers. J Appl Biomater Biomech.

