# Correction to: Gummy Smile: Mercado-Rosso Classification System and Dynamic Restructuring with Hyaluronic Acid

**DOI:** 10.1007/s00266-021-02229-z

**Published:** 2021-05-03

**Authors:** Jorge Mercado-García, Paula Rosso, Mar Gonzalvez-García, Jesús Colina, José Manuel Fernández

**Affiliations:** 1 Clinicas Jorge Mercado, Madrid, Spain; 2 Self Clinica, Barón de Finestrat 4, 03001 Alicante, Spain; 3 Centro médico estético Lajo-Plaza, Madrid, Spain; 4 Mar Gonzálvez-García, UCAM, Murcia, Spain; 5 Clínica Dr. Colina. Bilbao, Bilbao, Vizcaya Spain; 6 Centre Médic I D’Estética, Barcelona, Spain

## Correction to: Aesth Plast Surg 10.1007/s00266-021-02169-8

The article Gummy Smile: Mercado-Rosso Classification System and Dynamic Restructuring with Hyaluronic Acid, written by Mercado-García et al., was originally published electronically on the publisher’s internet portal on February 22, 2021 without open access. With the authors’ decision to opt for Open Choice the copyright of the article changed on March 11, 2021 to © The Author(s) 2021 and the article is forthwith distributed under a Creative Commons Attribution, which permits use, sharing, adaptation, distribution and reproduction in any medium or format, as long as you give appropriate credit to the original author(s) and the source, provide a link to the Creative Commons licence, and indicate if changes were made. The images or other third party material in this article are included in the article’s Creative Commons licence, unless indicated otherwise in a credit line to the material. If material is not included in the article’s Creative Commons licence and your intended use is not permitted by statutory regulation or exceeds the permitted use, you will need to obtain permission directly from the copyright holder. To view a copy of this licence, visit http://creativecommons.org/licenses/by/4.0/

